# Aptamers for the Delivery of Plant-Based Compounds: A Review

**DOI:** 10.3390/pharmaceutics16040541

**Published:** 2024-04-14

**Authors:** Joana Gamboa, Pedro Lourenço, Carla Cruz, Eugenia Gallardo

**Affiliations:** 1 Centro de Investigação em Ciências da Saúde, Universidade da Beira Interior (CICS-UBI), Av. Infante D. Henrique, 6201-506 Covilhã, Portugal; joana.gamboa@ubi.pt (J.G.); pedro.afonso.amaro.lourenco@ubi.pt (P.L.); 2 Departamento de Química, Faculdade de Ciências, Universidade da Beira Interior, Rua Marquês de Ávila e Bolama, 6201-001 Covilhã, Portugal; 3 Laboratório de Fármaco-Toxicologia, UBIMedical, Universidade da Beira Interior, EM506, 6200-000 Covilhã, Portugal

**Keywords:** aptamers, natural products, drug delivery systems

## Abstract

Natural compounds have a high potential for the treatment of various conditions, including infections, inflammatory diseases, and cancer. However, they usually present poor pharmacokinetics, low specificity, and even toxicity, which limits their use. Therefore, targeted drug delivery systems, typically composed of a carrier and a targeting ligand, can enhance natural product selectivity and effectiveness. Notably, aptamers—short RNA or single-stranded DNA molecules—have gained attention as promising ligands in targeted drug delivery since they are simple to synthesize and modify, and they present high tissue permeability, stability, and a wide array of available targets. The combination of natural products, namely plant-based compounds, with a drug delivery system utilizing aptamers as targeting agents represents an emerging strategy that has the potential to broaden its applications. This review discusses the potential of aptamers as targeting agents in the delivery of natural compounds, as well as new trends and developments in their utilization in the field of medicine.

## 1. Introduction

Natural products, which are derived from plants, animals, and minerals, have been used since the beginning of humanity [1]. The Chinese Herb Guide reports the use of herbal medicines as far back as 2000 BC [2].

Before the 20th century, natural products were the primary option for treating diseases in both animals and humans. However, in the 20th century, the idea of a drug–receptor interaction emerged, leading to a greater interest in pure and isolated compounds over extracts from natural products [1].

In the present era, there is a renewed interest in natural products as a reservoir of potential drug candidates, particularly for the treatment of cancer and immunosuppressive and neurological diseases, as well as for antihypertensive and anti-infectious purposes [3]. Despite the presence of alternative discovery methods, the unique properties of natural products continue to attract attention in the search for novel therapeutic agents [4]. They showcase extensive chemical diversity and complex structures, allowing them to demonstrate sophisticated bonding characteristics [2]. 

While natural products hold promise for therapeutic applications, their pharmacokinetic attributes are limited. The restricted solubility of these compounds, which leads to low absorption rates, hinders their bioavailability [5]. Some polyphenols, like curcumin and resveratrol, exhibit excessive lipophilicity, impeding their ability to dissolve effectively in the bloodstream. Conversely, highly hydrophilic compounds such as terpenoids and tannins face challenges in traversing biological membranes [6]. 

The use of natural products encounters obstacles attributed to pharmacokinetic and pharmacodynamic barriers, including issues such as insufficient solubility and bioavailability, among other inherent properties [7,8]. However, a promising approach for overcoming these hurdles is the encapsulation of these substances in nanoparticles. This strategy not only improves solubility but also holds the potential to elevate bioavailability. Additionally, nanoparticle encapsulation facilitates the targeted delivery of natural products, employing both passive and active mechanisms [9]. 

Active targeting consists of attaching a targeting ligand to the nanoparticle’s surface. In addition to improving bioavailability, it also enhances specificity by increasing the fraction of the drug that reaches the tissue of interest and decreases toxicity as the drug is released in a more particular area [6]. Aptamers are gaining popularity as targeting agents. They consist of DNA or RNA oligonucleotide sequences typically ranging from 25 to 80 bases and are recognized as promising targeting agents due to their distinctive attributes, such as remarkable stability, endurance under room temperature conditions, and resistance to multiple denaturation [10]. With a prolonged shelf life, these are cost-effective to synthesize, requiring only 2–8 weeks while maintaining consistent quality across batches [11]. They also exhibit high affinity and specificity, offering a wide range of targets, from small molecules to entire cells. Moreover, aptamers are relatively small compared with other targeting agents, such as antibodies [12].

Many researchers have chosen to employ a dual strategy, encapsulating natural products in nanoparticles and utilizing aptamers as targeting agents. The main goal of this review is to highlight the newly developed aptamer-based drug carriers and their clinical applications. Additionally, it discusses the use of natural compounds in combination therapy with other medicines, also applying aptamers as targeting agents.

## 2. Sources of Natural Products

The sources of natural products are diverse, encompassing plants, animals, marine organisms, and microorganisms (bacteria and fungi). These compounds exhibit a broad spectrum of bioactive properties, demonstrating their potential therapeutic applications. They include anti-inflammatory [13], antidiabetic [14], antiproliferative [2,15], anticancer [5,15,16], antimicrobial [17,18], antiprotozoal [19], antioxidant [20], antihyperlipidemic [2], antiasthmatic, and anti-obesity effects [21]. Additionally, they have beneficial effects on the cardiovascular system, acting as anti-arrhythmic and antihypertensive agents [22], as well as providing cardioprotective benefits [23]. Some natural compounds also exhibit sedative and antidepressant effects [2,24].

### 2.1. Natural Compounds from Plants

Besides the essential primary metabolites crucial to their development, plants also produce secondary metabolites [25,26]. These secondary metabolites are characterized by small molecular sizes, diverse chemical structures, and a wide range of chemical and biological activities [26]. These metabolites are related to the adaptation capacity and survival of plants, being produced as a form of communication and defense against predators or environmental factors. The type and quantity of secondary metabolites produced depend on external factors, such as temperature, nutrient deficits, and the amount of ultraviolet light, among others [25].

Lahlou et al. (2013) reported that about 40% of medicinal products are of natural origin or semi-synthetic derivatives, mainly from plants [1,26]. Yeshi et al. (2022) mentioned that 25% of known drugs are derived from the secondary metabolites of plants [26]. Morphine (isolated from *Papaver somniferum*), digitoxin (*Digitalis purpurea*), Taxol (*Taxus baccata*), artemisinin (*Artemisia annua*), quinine (*Cinchona officinalis*), vinblastine and vincristine (*Catharanthus roseus*), and aspirin (isolated as salicylic acid from *Filipendula ulmaria*) are landmark secondary metabolites isolated from plants [26].

The three groups of secondary metabolites based on their biosynthetic pathway and structure are phenolic compounds, terpenes, and nitrogen-containing compounds (with a special focus on alkaloids) [27].

#### 2.1.1. Alkaloids 

Alkaloids are secondary metabolites produced mainly as a defense against pathogens, insects, and animals, and they are found in seeds, roots, stems, and leaves of higher plants [15]. There are about 600 bioactive alkaloids [26] with anticarcinogen [28], antifungal [29], analgesic [30], antimalarial [31], anti-inflammatory [32], and antidiabetic potentials [33]. Alkaloids have a wide range of clinical applications, namely as anticancer (vincristine, berberine, camptothecin, vinblastine), antimalarial (quinine), and analgesic (morphine) agents [2,15], among others.

#### 2.1.2. Phenolic Compounds

In plants, phenolic compounds possess the ability to neutralize free radicals and filter harmful UV radiation. This capability aids them in adapting to extreme climates, such as heat or cold, and plays a role in reproductive processes [26].

Polyphenols encompass a wide range of natural products, including flavonoids, phenolic acids, xanthones, stilbenes, lignans, lignins, and tannins [24,26]. Flavonoids are the most abundant and comprise seven sub-groups (flavones, flavonols, flavanones, isoflavonoids, flavan-3-ols or catechins, and anthocyanins) [24,26].

They have been studied for their potential health benefits [34], including antioxidant and anti-inflammatory properties [35,36,37], which are associated with various health advantages, such as cardiovascular protection [38], anticancer properties [35,39], anti-aging effects [40], and neuroprotection [41]. Polyphenols are known to interact with membrane transporters in cells, influencing the transport of substances across the cell membrane. In the context of anticancer agents, cellular resistance can be a significant challenge, reducing the effectiveness of these medications. Some studies suggest that polyphenols may play a role in sensitizing cancer cells to cytotoxic agents [42,43]. By inhibiting membrane transporters, they can increase the intracellular concentration of medications, potentially improving treatment efficiency [44].

#### 2.1.3. Terpenes and Terpenoids

Terpenes and terpenoids perform several important activities for the plant: they are antioxidants [45], helping to overcome oxidative stress caused by external factors [12], and play an important role in the plant’s resistance to diseases [46]. 

Given their prominent role in defense mechanisms, these compounds exhibit a broad spectrum of antimicrobial and antifungal activities [47]. They facilitate cell rupture, inhibit protein and DNA synthesis, and disrupt microbe multiplication and development while also interfering with their physiological and metabolic processes [46].

Terpenes have molecular structures composed of isoprene units (2-methylbuta-1,3-diene), which can be rearranged into cyclic structures [46]. The number of isoprene units is the primary determinant of the structural diversity of terpenes. They have a wide range of sizes, starting from single-unit hemiterpenes (5C), progressing to mono- (C10), sesqui- (C15), di- (C20), sester- (C25), tri- (C30), and tetraterpenes (C40), and extending to polyterpenes (>C40) [46,48]. Terpenes exhibit simple hydrocarbon structures, while terpenoids, a modified class of terpenes, include additional oxygen-containing functional groups [46,48]. 

Menthol, categorized as a monoterpene, along with two extensively utilized drugs, paclitaxel (classified as a diterpenoid) and artemisinin (sesquiterpene), are well-known examples of compounds within this class [22].

##### Top of Form

In general, terpenes and terpenoids have a wide range of activities, such as anticancer properties (paclitaxel, docetaxel, carvacrol, linalool); antibacterial (terpineol’s isomers, eugenol, carveol, citronellol, geraniol, carvacrol), antimalarial (artemisinin), anti-inflammatory (triptolide, limonene, α-terpineol, β-pinene, α-pinene), antihyperglycemic (stevioside), and antioxidant activity (α-pinene and α-terpineol); antiallergic effects (atractylone, citronellol, carvone); and cardioprotection [46,49,50].

## 3. Enhancing Therapeutic Potential: Overcoming Challenges in Natural Product-Based Drug Development

Natural products and their derivatives interact with a wide range of important pharmacological targets, being very relevant in the treatment of various diseases, including cancer [1,51]. Due to their therapeutic potential, these compounds represent a great part of new medicines under development [4]. However, the problems inherent to natural products cannot be ignored: they have low specificity for the target, with associated adverse effects, low solubility, and thus, reduced bioavailability, and a short half-life, as many are eliminated quickly [5,52,53].

To address these challenges, various conjugation technologies have been employed, including the conjugation of natural products with peptides, proteins, antibodies, and viruses. Notably, conjugation with nucleic acids, particularly functional DNA, has shown significant relevance in targeted drug delivery and synergistic chemotherapy and in reaching new therapeutic targets that were previously unattainable [52]. 

## 4. Aptamers: General Concepts

Aptamers, a novel class of high-affinity nucleic acid for proteins, emerged around 1990 [54]. These short, single-stranded molecules—whether DNA, RNA, or synthetic XNA—demonstrate a notable ability to selectively and strongly bind to target molecules [55]. These biomolecules, typically composed of 25 to 80 nucleotides, function similarly to antibodies in their specific targeting [9,29]. Unlike antibodies, aptamers offer advantages such as a shorter generation time, reduced manufacturing costs, greater modifiability, enhanced thermal stability, increased target potential, and, notably, the absence of batch-to-batch variability [55,56].

### 4.1. SELEX

Aptamers are generated by an in vitro molecular evolution method known as “Systematic Evolution of Ligands by EXponential enrichment” (SELEX), which can be conducted against a variety of target molecules or elements, such as small compounds, proteins, nanoparticles, or live cells [55].

SELEX, a widely adopted method for generating aptamers, involves the initial incubation of the target of interest with a pool of single-stranded random oligonucleotides [57]. The oligonucleotides library typically consists of 40–100 single-stranded random nucleotide sequences flanked by primer-binding sites at both ends [56,57]. The process of aptamer generation unfolds through several key steps: (i) generating a random library of 10^14^–10^16^ single-stranded oligonucleotides, (ii) incubating these oligonucleotides with the target, (iii) separating bound from unbound oligonucleotides, (iv) selecting specific oligonucleotides, and (v) amplifying them either through PCR (for DNA aptamers) or RT-PCR (for RNA aptamers), followed by the final characterization of the aptamer through sequencing [56,58]. Notably, RNA libraries have proven successful in SELEX, with distinct protocols compared with DNA SELEX. These differences include the necessity of protecting RNA from RNases, amplification by T7 RNA polymerase, and a reverse transcription step before PCR. Consequently, the 5′-primer used for RNA SELEX typically encodes a promoter for T7 RNA polymerase. This iterative process continues until the desired oligonucleotide (or aptamer) with high binding affinity is obtained, and once achieved, these desired clones undergo further optimization to maximize their functional efficacy [56,57,58].

In recent years, various approaches have emerged to enhance the reliability and efficiency of aptamer generation. By making some changes to the SELEX method, new approaches have been developed, such as Immunoprecipitation-Coupled SELEX (IP-SELEX), Capture-SELEX, Cell-SELEX, Capillary Electrophoresis-SELEX (CE-SELEX), Atomic Force Microscopy-SELEX (AFM-SELEX), and Artificially Expanded Genetic Information System-SELEX (AEGIS-SELEX). These innovative techniques represent advancements in the field, each tailored to specific requirements and offer distinct advantages in the reliable and efficient generation of aptamers [57].

### 4.2. Aptamer Structure

Aptamers are composed of single-stranded DNA (ssDNA) or RNA, having distinct three-dimensional structures.

A significant shift has occurred, with most newly discovered and applied aptamers being DNA-based [59]. This preference for DNA is attributed to its enhanced stability and the elimination of the reverse transcription step during amplification, simplifying the selection process [60]. 

Beyond conventional DNA and RNA aptamers, there is an increasing acknowledgment of aptamers derived from XNA, covering non-natural or chemically modified nucleic acids [59]. One such example, introduced in a recent study by McCloskey et al., presents a distinctive subtype of aptamer referred to as “threomers”, aptamers crafted using alpha-L-threofuranosyl nucleic acid (TNA) [61]. Another distinct category is peptide aptamers, comprising short amino acid chains that exhibit specific binding to ligands [62]. 

While DNA, RNA, XNA, and peptide aptamers remain the most prevalent, additional variations exist, including Locked Nucleic Acid (LNA) aptamers [63], functionalized aptamers [64], and chimeric aptamers [65]. 

This diversification increases the range of aptamer libraries by expanding access to various binding epitopes on proteins [59].

### 4.3. Structure and Affinity of Aptamers

Aptamers inherently form complementary base pairs due to their inclination toward secondary and tertiary structures. These structural arrangements are pivotal in defining the functional properties of aptamers, influencing their capabilities of precisely binding to specific targets, including metal ions, small organic molecules, larger molecules, peptides, proteins, and liposomes [66,67].

The secondary structures of aptamers encompass a diverse array, including internal loops, stems, pseudoknots, bulges, kissing complexes, tetra loops, hairpins, and G-quadruplexes (G4) [68]. Leveraging pre-structured libraries is a strategic approach for enhancing the likelihood of the successful selection of a desired structure [59]. For instance, the incorporation of guanine (G)-rich sequences in a library increases the probability of G4 formation [59]. These structures are identified by the arrangement of two or more stacks of four guanine bases, forming coplanar structures. Each set of four guanines constitutes a building block, commonly referred to as a G-tetrad, which is stabilized by Hoogsteen hydrogen base-pairing under physiological conditions. Additionally, stability is maintained through π–π interactions and the presence of positively charged monovalent cations and hold interest not only for their unique structure but also due to their crucial role in critical cellular processes such as DNA replication, DNA damage repair, transcription, translation, and epigenetic modifications [69,70]. Given their frequent occurrence in high-affinity aptamers, elevating their representation in the starting library augments the chances of success.

Additionally, aptamers, empowered by their tertiary structures, possess the ability to distinguish between conformational isomers, recognize distinct epitopes of a target molecule, identify amino acid mutations, and differentiate various functional groups, even in closely related targets [67].

Aptamers can specifically bind to their targets via different forces, such as base stacking of aromatic rings, hydrogen bonding, van der Waals forces, complementarity in geometrical shape, and electrostatic interactions, collectively contributing to the binding affinity and specificity of the aptamer [67,68].

This multifaceted recognition capability underscores the versatility and precision of aptamers in various molecular interactions.

### 4.4. Aptamer Applications

Aptamers exhibit diverse applications because of their high affinity for specific target molecules, facilitating precise molecular recognition. The ease of engineering and customization further enhances their adaptability across various domains [71]. These versatility and cost-effective synthesis methods position aptamers as valuable tools in biomedical diagnostics, therapeutics, and diverse scientific disciplines, contributing to their widespread impact [72].

In the realm of biomedical diagnostics, aptamers find utility in detecting disease biomarkers and stem cell markers and aiding in cancer diagnosis, as exemplified by Miranda et al. through the application of the modified aptamer AS1411 for detecting nucleolin and its expression on the membrane of prostate cancer cells and in the peripheral blood mononuclear cells (PBMCs) of prostate cancer patients [73]. Moreover, their application extends to monitoring environmental contamination, showcasing their inherent capabilities in biosensor technology [56,57].

Beyond diagnostics, aptamers play a pivotal role in therapeutics [56]. They can either activate or inhibit specific targets [57], showcasing their potential in modulating biological processes. Furthermore, aptamers excel in enabling targeted drug delivery, contributing to advancements in precision medicine [55].

The previously mentioned AS1411, a DNA aptamer, has emerged as one of the most extensively studied aptamers, showcasing its versatile applications in diagnostics and therapeutics and as a valuable tool in biomedical research [74,75]. AS1411 is a 26-base oligodeoxyribonucleotide aptamer rich in guanine, forming G4 structures, and is renowned for its unique ability to bind to nucleolin [75]. AS1411 aptamer exhibits a polymorphic secondary structure. Changes in conditions, such as rapid cooling, alter the kinetic and dynamic properties, resulting in different conformations. Moreover, even under the same experimental conditions, it can adopt several distinct monomeric conformations, and multiple conformations may coexist simultaneously [76]. By targeting cells with elevated nucleolin concentrations, a common feature in many cancer cells, AS1411 has progressed to phase II clinical trials for acute myeloid leukemia and renal cell carcinoma [77,78]. Its antiproliferative effects stem from interactions with nucleolin, along with additional nucleolin-independent mechanisms [75]. AS1411 also serves as a carrier for cancer-selective drug delivery, underscoring its versatility and promising potential in therapeutic applications [79].

#### Aptamer-Based Drug Carriers for Delivery

The application of aptamers in targeted drug delivery has been explored, particularly in delivering therapeutic agents such as chemotherapeutics, small interfering RNAs (siRNAs), microRNAs (miRNAs), toxins, and more [80]. Aptamers, serving as targeting ligands, are often encapsulated into nanoparticle platforms, which act as carriers for the therapeutic payload [81]. This approach has been applied across various therapeutic modalities, encompassing chemotherapy, immunotherapy, and beyond [82]. The formation of these aptamer–nanoparticle systems involves diverse methods, commonly featuring the direct attachment of aptamers to drugs using various cleavable or non-cleavable linkers [80,83]. It is noteworthy that while aptamers contribute to the targeting specificity, the enhanced solubility and bioavailability of natural products and other therapeutic agents are primarily derived from the nanoparticle component of these delivery systems [84].

Aptamers, when combined with nanomaterials, create effective delivery systems in bioanalysis and biomedicine [80,83]. Nanomaterials, with unique properties like small size (optimal being below <100–200 nm [85]) and high loading capacity, overcome the limitations of traditional approaches [80,86]. Advancing nanomedicine hinges on improving the precise identification of diseased tissues, and the synergistic partnership between aptamers and nanomaterials shows potential for targeted drug delivery [87,88].

Various nanomaterials, both inorganic (e.g., gold nanoparticles, silica nanoparticles, carbon nanomaterials) and organic (e.g., liposomes, micelle structures, DNA hydrogels), offer unique properties for biomedical applications. Inorganic options provide high surface-to-volume ratios and controlled drug release, while organic nanomaterials, such as liposomes and micelle structures, demonstrate biocompatibility and efficient drug loading. Target-responsive DNA hydrogels, among aptamer-based organic nanomaterials, stand out for their mechanical properties and programmable features, making them valuable in biomedical and pharmaceutical applications [80,88].

**Figure 1:**
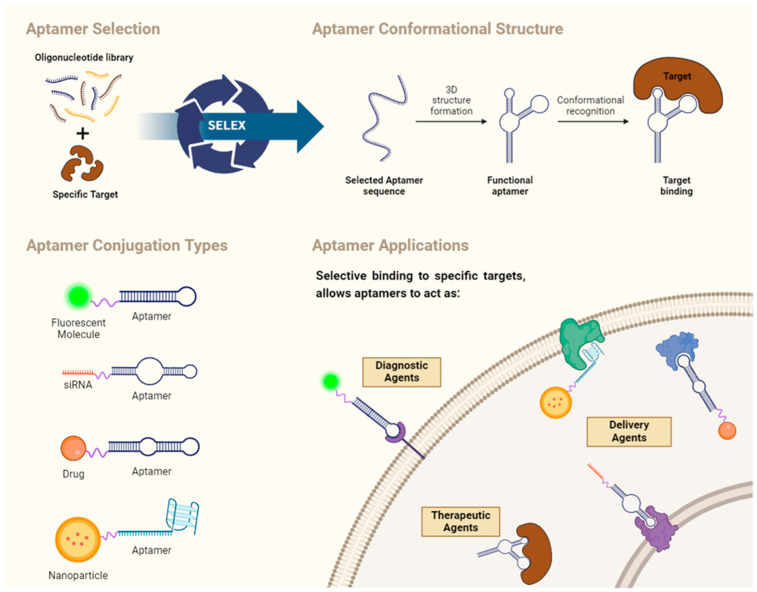
Schematic illustration of the lifecycle of aptamers, from their genesis through the SELEX method to practical applications. Aptamers, honed for specific targets, derive their binding specificity from a unique structure. Initially crafted for target binding, aptamers offer direct utility—either by inhibiting or activating their targets. Their adaptability extends with the option to enhance diagnostic capacities or serve as drug-delivery agents through the conjugation with additional molecules. This array of options positions aptamers as versatile tools across diverse biomedical processes. Their journey, depicted in this figure, highlights their significance in biomedical research and applications.

In Figure 1, a comprehensive overview of key aspects pertaining to aptamers, including their selection processes, structural features, types of conjugation, and diverse applications/functions, is presented based on the discussions outlined in this paper.

By combining the precision of aptamer drugs for targeted drug delivery with the latest advancements in natural products, aptamers, and delivery systems, we are forging a pathway toward more refined and effective therapeutic interventions. This synergy not only enhances the specificity of drug delivery but also taps into the unique properties of natural products, offering a comprehensive and promising strategy for advancing therapeutic approaches.

## 5. Current Plant-Based Natural Products, Aptamers and Delivery Systems

Combining the strengths of aptamers with the inherent challenges of plant-based natural products, a diverse array of studies has surfaced. In these investigations, natural compounds are strategically loaded into nanoparticles and modified with aptamers, presenting a promising approach to overcoming inherent limitations, such as solubility, stability and bioavailability, and enhance the efficacy of these compounds in permeating and accumulating in tissues with great precision [87]. This innovative synergy not only mitigates the challenges associated with natural products but also elevates their potential for various applications.

Thus, a comprehensive literature search was carried out on the PubMed and Web of Science databases, encompassing English-language publications from 1 January 2018 to 31 December 2023. The search terms employed on PubMed were “Aptamers, Nucleotide”[Mesh] AND “Drug Delivery Systems”[Mesh], leading to 336 articles. In the Web of Science, the terms “Aptamers (Topic)” AND “Drug Delivery (Topic)” were employed, resulting in the initial retrieval of 397 articles. Among these findings, all articles addressing delivery systems developed to deliver plant-based natural compounds using aptamers as targeting agents were selected. The screening process involved the evaluation of titles, abstracts, keywords, or content, leading to the inclusion of 43 articles. The results found were subdivided into two sub-groups: natural compounds extracted from plants, with known therapeutic potential but with low expression in clinical practice, and plant-based semi-synthetic drugs widely used. The emphasis and level of detail were greater for the first group because it is a less explored topic.

### 5.1. Alkaloids Based Aptamer-Carriers

#### Homoharringtonine

Homoharringtonine (HHT), a natural alkaloid from *Cephalotaxus harringtoni*, exhibits potent anticancer properties. It suppresses cell growth and viability and induces apoptosis via the dysregulation of the mitochondrial function—a known trigger for programmed cell death [89]. In lung cancer, HHT has demonstrated inhibitory effects on the proliferation of Gefitinib-resistant non-small cell lung cancer (NSCLC). Moreover, HHT exerts its anticancer effects by inhibiting the phosphorylation of STAT3 through the JAK/STAT3 pathway, which typically promotes pro-survival and pro-proliferative signals in NSCLC cells. In these cancer cells, the epidermal growth factor receptor (EGFR) is usually upregulated, being a prominent target. This overactivation leads to the uncontrolled activation of anti-apoptotic signaling pathways and unrestricted cell proliferation [90].

In order to target lung cancer cells, Zhang et al. developed polymeric nanoparticles (POL) loaded with HHT and functionalized them with the EGFR aptamer. To achieve this, poly(L-lactide-co-glycolide)-S-S-polyethyleneglycol (PLGA-SS-PEG) was prepared via two-step polymerization and loaded with HHT using a solvent-evaporation approach. The EGFR amino-modified aptamer was bound to the PLGA-SS-PEG carboxylic group through the EDC/NHS method [91]. In vitro cytotoxicity was assessed using Beas2B (human normal lung epithelial cells), A549 (human lung adenocarcinoma cells), and NCI-H226 (human lung squamous cell carcinoma). In vivo experiments were performed to evaluate the efficacy of aptamer-modified and untargeted formulations on the reduction of tumor growth in mice injected with A549 human lung carcinoma cells. Fluorescence microscopy analysis indicated that the EGFR aptamer-functionalized nanoparticles effectively targeted lung cancer cells (A549 and NCI-H226) over normal lung cells (Beas2B). The IC50 of HHT was lower with the Apt-HHT-POL formulation than with free HHT both in A549 and NCI-H226 cells, indicating higher efficacy. Furthermore, Apt-HHT-POL induced apoptosis and inhibited tumor growth more effectively while exhibiting fewer toxic effects on liver function compared with free HHT, as evaluated using AST and ALT levels, which were increased with the free anticancer drug [91].

### 5.2. Phenolic Compound-Based Aptamer Carriers

#### 5.2.1. Apigenin

Apigenin, a flavonoid found in certain vegetables, fruits, herbs, and plant-based beverages, exhibits diverse properties, including antioxidant effects and potential therapeutic applications in autoimmune, neurodegenerative, and inflammatory diseases [92]. Its anticancer potential has also been described [92,93]. Both in vitro and in vivo studies have shown that apigenin promotes apoptosis, induces cell cycle arrest, suppresses cancer cell invasion, and exhibits autophagy and immunogenic activities [93]. Apigenin is capable of causing cell cycle arrest at various proliferation stages, including the G1/S phase or G2/M phase, through the modulation of different cyclin-dependent kinases (CDKs) and other genes. Additionally, apigenin increases the levels of p53 (tumor suppressor protein) and the p53-induced gene products, regulating a p53-Bax-caspase-3 apoptotic pathway [92]. Despite its numerous advantages, poor specificity, solubility, and bioavailability limit their use in vivo studies [92,93]. 

Dhara and colleagues developed aptamer-functionalized PEGylated nanoliposomes to deliver apigenin to cancer cells in rats with hepatocellular carcinoma. The amino-modified AS1411 aptamer, which exhibits affinity to nucleolin overexpressed on the surface of hepatocellular carcinoma cells, was employed in their study [93]. In an initial phase, apigenin was encapsulated in pegylated nanoliposomes (PEG-LP) using the thin-film hydration method [54,55,94]. The functionalization of PEG-LP with the aptamer was achieved through covalent linking, where the amino group of the aptamer (NH2-modified AS1411) was attached to the carboxyl group of PEG-LP [93,95]. Aptamer-based liposomes showed a higher cytotoxic potential when compared to PEGylated liposomes alone and free apigenin. Nanoliposomes also showed a higher percentage of apoptosis (85.6%) when compared to PEGylated liposomes (74%). The higher apoptotic levels observed when using the aptamer nanoformulation are explained by an increased expression of p53 and caspase-3 (which plays an important role in apoptosis) and a reduction in Bcl-2 (anti-apoptotic proteins) expression. They also noticed that the aptamer nanoconjugate accumulated more in the liver than in other tissues [93].

The improvement in bioavailability and precise delivery through this functionalized nanoformulation increased the apoptotic potential of apigenin and, thus, its antitumoral effect, even at low doses [93].

#### 5.2.2. Curcumin 

Curcumin is a natural polyphenol present in *Curcuma longa* (turmeric), widely used in spices, especially in Asian countries, because of its flavor [96]. It exhibits anti-inflammatory, anticancer, and antioxidant properties [14]. It can impact various biological targets, including transcription factors, growth factors, inflammatory mediators, cytokines, cell cycle proteins, enzymes, protein kinases, and apoptotic proteins [59]. Additionally, it influences crucial cellular pathways related to cell survival, tumor suppression, caspase activation, and death receptor pathways [60]. The anticancer properties of curcumin are attributed to its multifaceted impact on various cellular mechanisms [97,98]. It downregulates activator protein-1 (transcription factor associated with anti-apoptotic, mitogenic, and pro-angiogenic genes), suppresses the PI3K/Akt signaling pathway, induces G0/G1 phase arrest, and downregulates the STAT3 pathway [97]. The versatility of curcumin extends its efficacy to different cancer types [97]. It also shows antidiabetic, anti-macrobian, anticancer, and anti-aging effects, as well as demonstrating efficacy in wound healing, arthritis, and Alzheimer’s [59]. Because of its phenolic composition, curcumin has antioxidant powers since it eliminates various forms of reactive oxygen species (ROS) [98]. However, the practical application of curcumin is constrained by issues like limited solubility, inadequate bioavailability, instability under physiological conditions, and a brief half-life in the gastrointestinal (GI) tract [96,99,100]. To overcome these difficulties, some authors opt to use curcumin-encapsulated nanoparticles, improving their solubility and bioavailability [63,101].

Ma et al. developed curcumin liposomes (CUR-LPs) and used the A15 aptamer for the active targeting of prostate cancer cells (DU145 cell line). A15 aptamer has been proven to be a promising ligand for targeting CD44+ and CD133+ cells [102]. This aptamer comprises 15 oligonucleotides and exhibits a predicted hairpin secondary structure [103].

CD44, an adhesion molecule implicated in tumor migration, progression, and metastasis, and CD133, a cell surface glycoprotein of uncertain function but identified in human solid tumors associated with aggressive behavior and metastasis, serve as markers for cancer stem cells (CSCs) [104]. 

The investigation conducted by Ma et al. involved a comparative analysis of free curcumin, CUR-LPs, and A15-CUR-LPs, focusing on hemolytic potential, cytotoxicity, and intracellular drug concentration [102]. A15-CUR-LPs were synthesized in two main steps. Initially, CUR-LPs were prepared using the thin-film dispersion method. Then, aptamer conjugation to curcumin liposomes was accomplished through a thiol–maleimide reaction [102,105]. A15-CUR-LPs demonstrated a hemolytic potential equivalent to that of CUR-LPs and lower than that of free curcumin, establishing the safety of the formulation for injection [102]. Regarding cellular cytotoxicity, both CUR-LPs and A15-CUR-LPs exhibited increased intracellular accumulation, leading to an enhanced cytotoxic potential that was initially comparable. However, over an extended incubation period, A15-CUR-LPs demonstrated a more pronounced inhibitory effect on cancer cells, indicating a superior performance in terms of sustained and potent anticancer activity. The selectivity for tumor tissue was also higher with A15-CUR-LPs, as well as this formulation being the one that led to the highest reduction in tumor size [102]. 

Alibolandia et al. loaded pegylated poly(amidoamine) (PAMAM) G5 dendrimers with gold nanoparticles (AuNPs) and curcumin, creating PEG-AuPAMAM-CUR. Subsequently, they adorned these constructs with the mucin-1 (MUC-1) aptamer, resulting in Apt-PEG-AuPAMAM-CUR [106]. To achieve that, first, curcumin was mixed with a PEG-coated AuPAMAM dendrimer solution, and then a thiolated MUC-1 aptamer was covalently attached to AUPAMAM nanoparticles [106].

There are several aptamers targeting MUC-1 that can adopt secondary structures typical of single-stranded DNA oligonucleotides. In this instance, an aptamer comprising 35 oligonucleotides was used [107]. MUC-1, a transmembrane glycoprotein, is typically expressed in various epithelial cells, including those in the mammary gland, esophagus, stomach, duodenum, pancreas, uterus, prostate, and lungs, as well as in hematopoietic cells. Its aberrantly glycosylated form is often overexpressed in various epithelial cancers, such as colorectal, breast, lymphocytic leukemia, adrenal cell carcinoma, and prostate carcinoma [108]. AuNPs were employed primarily for imaging (diagnosis) purposes. Alibolandia and colleagues conducted tests in mice using free curcumin, PEG-AuPAMAM-CUR, and Apt-PEG-AuPAMAM-CUR. Notably, Apt-PEG-AuPAMAM-CUR exhibited the most promising results, particularly in reducing tumor volume after eighteen days post-injection and enhancing the survival rate of mice when compared to PEG-AuPAMAM-CUR and free curcumin [106]. Additionally, it demonstrated elevated cellular uptake, internalization, and cytotoxicity in C26 and HT29 colorectal cancer cells, surpassing the performance of PEG-AuPAMAM-CUR [106]. 

In another study involving curcumin, Saleh and colleagues formulated a curcumin-loaded human serum albumin (HSA) nanoparticle with HER2 (human epidermal growth factor receptor-type 2) aptamer (Apt-HSA-CUR-NP) [109]. To accomplish this, curcumin was loaded into HSA nanoparticles using albumin-bound technology [109,110]. Then, the carboxylic groups of HSA were activated and covalently bound to the amino group of the aptamer through an EDC/NHS reaction to form the conjugated Apt-HSA-CUR-NP [109,111]. HER2 is a tyrosine kinase receptor related to cell proliferation, migration, invasion, and survival and is overexpressed in some breast carcinomas. Normally, the expression of HER2 in breast cancer is associated with poor prognosis; however, this expression allows targeted anticancer therapy [112]. To test the efficacy and safety of the targeted delivery of Apt-HSA-CUR-NP to HER2-positive breast cancer cells, they used two cell lines, one HER2-positive (SK-BR3) and one HER2-negative (MCF-7). They showed that the use of the aptamer increased the HSA-CUR-NP uptake by HER2-positive cells (SK-BR3), which suggests a good targeting capacity [109]. In terms of cytotoxicity, the impact of Apt-HSA-CUR-NP surpassed that of both free curcumin and HSA-CUR-NP at equivalent concentrations. This was evident as the viability of SK-BR3 cells exhibited a notably greater reduction in the presence of the aptamer-based formulation. Furthermore, in cells that do not express HER2 (MCF-7), the Apt-HSA-CUR-NP formulation was less cytotoxic, showing that it may have fewer side effects [109].

As previously mentioned, various formulations of curcumin have been devised to enhance its characteristics. Numerous researchers have engineered delivery systems incorporating curcumin, which have been extensively discussed in multiple reviews on the subject. These reviews, such as those by Sanjanwala et al. [113] and Tong et al. [77], focus on targeted drug delivery in cancer and highlight systems using the AS1411 aptamer. Additionally, the works of Alshaer et al. [114], Fu et al. [115], and Hu et al. [116] cover various applications of aptamers and aptamer-modified nanoformulations, mentioning several drug delivery systems loading curcumin.

#### 5.2.3. Epigallocatechin Gallate

Sheikh et al. conducted a review on aptamer-chitosan nanoparticles for cancer therapeutics, where they mentioned a plant-based natural compound—epigallocatechin gallate (EGCG) [117]. EGCG is the most abundant polyphenolic compound in green tea, and it is also found in other plants [118]. Renowned for its potent antioxidant properties [118,119], EGCG offers various health benefits, including anti-inflammatory, neuroprotective [120], and anticancer effects [121]. In cancer therapy, EGCG inhibits cell proliferation and tumor growth, induces apoptosis and cell cycle arrest, suppresses invasion and metastasis, and hinders angiogenesis [122].

#### 5.2.4. Genistein

Rotoli et al. conducted a review on the applications of aptamers for targeting NSCLC [123], where they mentioned a delivery system loading genistein. Genistein, a natural compound classified as an isoflavone, belongs to a group of phytoestrogens found in soybeans and other leguminous plants [124]. It inhibits lung cancer cell growth by downregulating essential oncoproteins like pAKT and p-PI3. The dysregulation of these signaling pathways, often through the overactivation of pAKT and p-PI3K [125], can contribute to tumor growth, progression, and resistance to treatment [126].

#### 5.2.5. Mangosteen

α-mangosteen is the main xanthone found in mangosteen, a fruit from the mangosteen tree (*Garcinia mangostana*). It exhibits antioxidant, anti-inflammatory, antibacterial, antifungal and anticancer activities [127]. The anticancer potential of mangosteen is associated with various molecular pathways related to the control of cell growth and survival. It reduces the expression of pro-invasive molecules, such as MMP-2 and MMP-9, suggesting a potential impact on the invasive capability of tumor cells. Concurrently, there is an increase in pro-apoptotic molecules, including p53, Bax (a pro-apoptotic protein), and caspase activity (3, 7, 8, and 9), indicating a promotion of programmed cell death [128]. Additionally, the negative regulation of hyperactive cellular signaling pathways in cancer, associated with cell growth and survival, along with cell cycle arrest in the S/G2/M phases, demonstrates its antitumoral capacity. These combined effects highlight its potential as an agent in cancer therapy.

Mangosteen exhibits promising potential as an adjuvant in cancer therapy, particularly in targeting multicellular tumor spheroids (MCTSs), three-dimensional structures that mimic in vivo tumors. It has demonstrated activity against MDA-MB-231 human breast cancer cell-generated MCTSs, resulting in disaggregation, reduced overall tumor bulk, and decreased cell viability [127].

To test the efficacy of α-mangosteen, Bonafé and colleagues used multicellular tumor spheroids generated from a breast cancer cell line (MCF-7). This group encapsulated α-mangosteen in lipidic nanoparticles to improve the selectivity for tumor cells. Since these cells highly express CD44, they conjugated an α-MG-loaded NP with a CD44 thioaptamer and evaluated it against c without conjugation [129]. To synthesize the combined lipid-polymer nanoparticles, (poly(lactic-co-glycolic acid) PLGA, soy lecithin, and 1,2-diasteroyl-glycero-3-phosphoethanolamine-N-carboxy (polyethylene glycol)2000 (DSPE-PEG2000-COOH) were used. Mangosteen was incorporated into the PLGA. Then, the carboxylic group of DSPE-PEG2000-COOH was covalently bound to an amino group of aptamer through EDC/NHS protocol [129,130].

A-MG-loaded NPs increased the distance between cells not only in the peripheral layers but also in the interior of spheroids. The concentration of α-MG that significantly reduced spheroid density and induced the most effective disaggregation was approximately one-tenth lower than the concentrations causing damage to MCF-7 MCTSs treated with free α-MG. Therefore, their conclusion suggests that NPs are effective carriers for targeting tumors at minimal concentrations and low doses. Consequently, α-MG-loaded NPs emerge as potential non-toxic adjuvants for tumor chemotherapy that merits further investigation. They also may improve both the penetration and diffusion of conventional drugs into the tumor bulk because of their disaggregation power [129].

It was observed both the reduction and disaggregation of tumor spheroids when α-MG-loaded NPs conjugated to the CD44 thioaptamer were used, indicating an enhanced efficacy of this aptamer-modified approach. Additionally, the clearance or removal of the conjugated NP from the system was found to be lower than the unconjugated NP, suggesting a long retention time and potential sustained therapeutic impact. This enhanced efficacy and prolonged retention make the α-MG-loaded NP conjugated to the CD44 thioaptamer a promising candidate for further exploration in cancer therapy [127].

#### 5.2.6. Morin

Morin is a secondary metabolite found in figs and other Moraceae plants that is known for its anticancer [131], antioxidant, and significant anti-inflammatory properties [132]. Despite its potential for various applications, its effectiveness is hindered by its low water solubility and bioavailability [133].

Ding et al. developed liposomes encapsulating Morin functionalized with Au–Apt nanoparticles. The AS1411 aptamer with a disulfide modification was incubated with Au nanoparticles to form Au–Apt nanoparticles [134]. In vitro, the cytotoxic and apoptotic effects were tested using SGC-7901 cells (human gastric cancer cell line). Tumor growth in vivo was evaluated in mice using an SGC-7901 tumor xenograft model [134].

The Apt-AU@morin-LP formulation exhibited high cytotoxicity, inducing structural alterations in SGC-7901 cells. This formulation has demonstrated selectivity toward these cells, with low toxicity observed in normal cells. Cells treated with free Morin showed an apoptosis ratio of 3.51%. Apt-AU@morin-LP at various concentrations showed increased apoptotic activity, with apoptosis ratios of 7.44%, 10.75%, 15.53%, and 40.77% at increasing concentrations, accompanied by a gradual decrease in viable cells and cell density. This result confirms the effective apoptotic induction by Apt-AU@morin-LP in tumor cells. Furthermore, mice treated with Apt-AU@morin-LP showed a notable reduction in tumor weight and size. Importantly, mice receiving Apt-AU@morin-LP treatment exhibited significantly prolonged survival compared with the other groups [134].

### 5.3. Terpenes and Terpenoids Based Aptamer Carriers

#### 5.3.1. Celastrol

Celastrol is a pentacyclic triterpenoid derived from the medicinal plant *Tripterygium wilfordii* and is renowned for its great anticancer potential [135]. Recognized as a potent anticancer agent, it exerts its influence through distinct mechanisms. It promotes the downregulation of the PI3K/Akt/mTOR pathway, crucial for regulating cellular processes such as growth, proliferation, survival, and metabolism, along with the Wnt/β-catenin pathway, which regulates various phenomena during embryonic development, organogenesis, and differentiation [136,137]. These pathways are frequently hyperactivated in cancer. Additionally, it downregulates the STAT3 protein, another component often hyperactivated in cancer and associated with cellular proliferation and survival, and inhibits vascular endothelial growth factor (VEGF)-induced vasculogenesis, crucial for preventing the formation of new blood vessels. On the other hand, celastrol activates the Bcl-2/Bax-caspase 9/3 cascade and the ROS/JNK pathway, leading to programmed cell death and apoptosis. Finally, it induces autophagy through the AR/miR-101 pathway, contributing to its comprehensive efficacy as an anticancer agent [137]. However, its challenges are blood stability, toxicity, and some side effects [137]. To improve these features, Niu and colleagues developed a delivery system composed of PEGylated G5 PAMAM dendrimers and an EpCAM aptamer to target moiety to deliver celastrol (Apt-Ce-PAMAM) to colorectal cancer cells. Moreover, they also developed another delivery system for celastrol but with an antibody as a targeting agent. 

To develop Apt-Ce-PAMAM, G5-PAMAM dendrimers were carboxylated to create G5-COOH dendrimers. These dendrimers were then pegylated by using the carboxyl groups to react with the amine groups of PEG (NH2-PEG-COOH) [135,138]. Subsequently, EpCAM aptamers were conjugated to the surface of PEGylated PAMAM dendrimers using the EDC (1-ethyl-3-(3-dimethylaminopropyl)-carbodiimide)/NHS(N-hydroxysuccinimide) protocol [61,64,65]. Finally, the drug was covalently bound to the residual amine groups of PAMAM dendrimers using the EDC catalytical method [135,138,139]. They not only proved the efficacy of PEGylated dendrimers in the delivery of this medicine to cancer cells but also showed a higher accumulation of aptamer nanoconjugates in tumor sites compared with antibody nanoconjugates. Aptamer nanoconjugates also had a superior intratumoral penetration capacity, and therapeutic efficiency was 20% higher compared with antibody nanoconjugates. The anticancer efficiency of celastrol delivered by PEGylated aptamer dendrimers at 2 mg/kg was above 92%, demonstrating significant efficacy with no apparent side effects [135].

#### 5.3.2. Thymoquinone

Thymoquinone, a monoterpene derived from *Nigella sativa* seeds, exhibits antineoplastic activity with a particular focus on breast, liver, and colon cancers [140]. In vivo and in vitro studies have demonstrated that the anticancer activity of thymoquinone is mediated via different mechanisms of action. It influences various biological pathways that are implicated in proliferation, cell cycle regulation, apoptosis, angiogenesis, carcinogenesis, and cancer metastasis [141]. It possesses additional health benefits, including antioxidant, cardioprotective, hypoglycemic, and anti-inflammatory properties [140]. Thymoquinone exerts its antioxidant effect by upregulating the mRNA expression and activation of antioxidant cytoprotective enzymes that play a crucial role in scavenging hydrogen peroxide and superoxide radicals, thereby preventing lipid peroxidation and mitigating the cellular damage caused by oxidative stress [141]. However, its highly hydrophobic nature has posed challenges in its application [142].

In a study by Murphy and colleagues, an innovative approach was developed using AS1411 nanodroplets loaded with thymoquinone to target breast cancer cell lines (MDA-MB-231 and HCC1395) [142]. Thymoquinone alone and the untargeted formulation were included for comparative analysis. Nanodroplets were formulated with perfluoropentane and lipids and arranged into a perfluorocarbon core surrounded by a lipid layer. Thymoquinone, being hydrophobic, was integrated into the lipidic solution. In the functionalized formulation, the AS1411 aptamer, modified with thiol groups, was bound to the lipid molecules of the nanodroplets via a thiol–maleimide reaction [142]. Thymoquinone demonstrated the ability to modulate the expression of the cytochrome P450 family, impacting estrogen metabolism. This modulation has potential implications for breast cancer, as estrogen-related pathways are linked to the development of this cancer type. Additionally, thymoquinone downregulated the expression of genes associated with estrogen and interferon pathways, further suggesting its potential to interfere with these pathways relevant to breast cancer [140].

The study’s findings indicated a notable difference in cell uptake between AS1411 nanoemulsion and untargeted nanoemulsion within the initial 4 h of incubation. However, over a longer period, both formulations exhibited substantial uptake by cancer cells, potentially limiting the specificity of the in vitro study due to non-receptor-mediated endocytosis. While AS1411 targeting showed a small, non-statistically significant increase in cytotoxicity in MDA-MB-231 cells compared with untargeted nanoemulsions, no such increase was observed in HCC1395 cells. Nevertheless, both targeted and untargeted nanoemulsions significantly increased the cytotoxicity of thymoquinone compared with the free compound alone in both cell lines. These results underscore the potential therapeutic value of nanoemulsions, especially when targeted, in enhancing the cytotoxic effects of thymoquinone in breast cancer cells [142].

#### 5.3.3. Triptolide

Triptolide, structurally a *diterpenoid epoxide*, is one of the active ingredients of the Chinese medicinal plant Thunder God Vine (*Tripterygium wilfordii* Hook. f.). It is known and used for its anti-rheumatic, antimicrobial, anti-inflammatory, immunomodulatory, and anticancer properties [51,143]. Its cytotoxicity is explained by its capacity to inhibit RNA polymerase II-mediated transcription and, consequently, inhibit cell activation and proliferation [51]. However, it presents high toxicity, poor solubility, and poor bioavailability, which limits its clinical use [143]. Since the major challenge with triptolide is to reduce its adverse effects and improve its pharmacokinetics, some strategies are being employed [51].

Nucleolin is also overexpressed in membranes of pancreatic cancer cells and Triple-Negative Breast Cancer (TNBC) cells; AS1411 can, therefore, be used to target these cells [143].

In a preclinical study conducted by He J et al., an AS1411−triptolide (ATC) conjugate was developed for the treatment of TNBC. To achieve that, triptolide was covalently linked to an amino-AS1411 aptamer through a C-N bond formation between phenylformate triptolide and the amino group of the aptamer [143]. The study revealed that the number of observed tumor cells at the end of the treatment was significantly lower for ATC, indicating higher efficiency in inhibiting TNBC tumor growth and inducing apoptosis. Furthermore, the investigation demonstrated that ATC caused no significant damage to major organs and exhibited less physical toxicity, emphasizing its potential as a promising therapeutic option for TNBC. Despite free triptolide showing negligible in vivo TNBC tumor inhibition at the same dosage level, the introduction of the AS1411 aptamer remarkably enhanced the in vivo antitumor activity of triptolide for TNBC treatment [143].

Additionally, several reviews have also discussed another therapeutic delivery system for chemotherapy-resistant pancreatic cancer involving triptolide. Sanjanwala et al. conducted a review on aptamers and nanobodies as ligands for targeted drug delivery in cancer, particularly mentioning drug delivery systems utilizing the AS1411 aptamer [113]. Yin et al. reviewed drug delivery systems using aptamers as functionalizing agents for therapeutics in various types of cancer [144], and Li et al. reviewed the application of aptamers as drug-delivery carriers related to adenocarcinoma [145]. These reviews mention the delivery system developed by Wang et al., specifically a micelle containing triptolide functionalized with the AS1411 aptamer, demonstrating the superior efficacy of the aptamer-functionalized micelle compared with the non-functionalized micelle in tumor growth in vivo [146].

**Figure 2:**
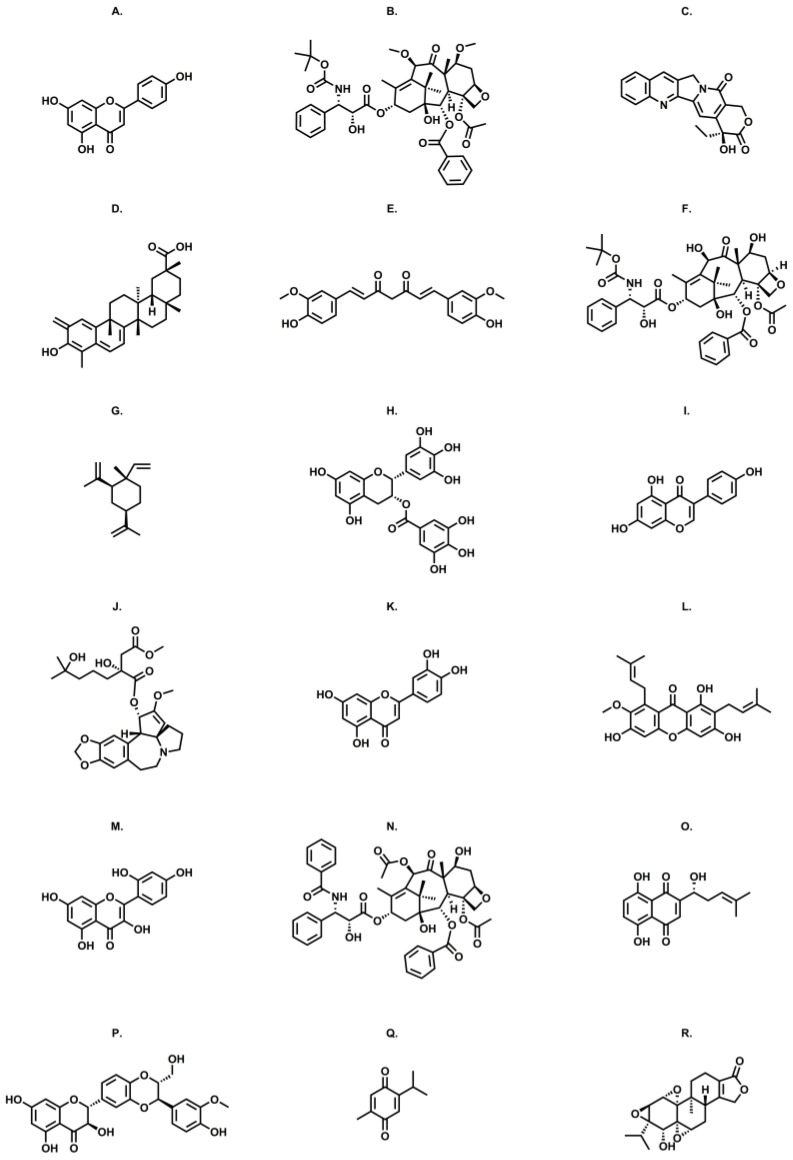
Chemical structures of plant-based natural compounds associated with aptamers in drug delivery systems: (**A**)—Apigenin; (**B**)—Cabazitaxel; (**C**)—Camptothecin; (**D**)—Celastrol; (**E**)—Curcumin; (**F**)—Docetaxel; (**G**)—β-elemene; (**H**)—Epigallocatechin gallate; (**I**)—Genistein; (**J**)—Homoharringtonine; (**K**)—Luteolin; (**L**)—Mangosteen; (**M**)—Morin; (**N**)—Paclitaxel; (**O**)—Shikonin; (**P**)—Silibinin; (**Q**)—Thymoquinone; (**R**)—Triptolide.

Figure 2 summarizes the chemical structures of plant-based natural compounds associated with aptamers in drug delivery systems.

Nanoparticles have the capability to enhance the pharmacokinetic properties of natural products by improving their absorption and solubility and then increasing bioavailability. Aptamers enhance the specificity and efficacy of natural products by delivering them to specific targets, thereby reducing concerns related to toxicity. Consequently, the synergy between nanoparticles, aptamers and natural products overcomes the many limitations of natural compounds alone, expanding their therapeutic potential. Table 1 summarizes the latest advancements in aptamer-based drug carriers, offering a comprehensive overview of recently developed combinations.

### 5.4. Synergistic Therapy

Previously, we presented drug delivery systems designed to deliver plant-based natural compounds in monotherapy. At this point, we highlight drug delivery systems combining natural products with well-established chemotherapeutic agents widely used in clinical practice, many of which are also derived from natural sources.

#### 5.4.1. Curcumin

Curcumin has already been used in cancer therapy in combination with other drugs. Chen and colleagues [147] developed curcumin- and cabazitaxel-loaded PLGA-PEG nanoparticles, functionalized with the A10-3.2 aptamer for targeted prostate cancer therapy [147]. This aptamer, which targets PSMA, consists of 39 nucleotides in its sequence and exhibits a predicted hairpin secondary structure [153]. To prepare a functionalized formulation, the amino-modified A10-3.2 aptamer was conjugated to the carboxylic groups of lipid-polymeric nanoparticles (Apt-POL) through an EDC/NHS reaction [147,154]. Then, CTX, curcumin, and Apt-POL were dissolved in chloroform and subjected to nanoprecipitation [147,155].

Combined cabazitaxel and curcumin showcased a synergistic effect, demonstrating superior cell inhibition compared with the individual applications of cabazitaxel or curcumin. Moreover, the aptamer-conjugated nanoparticles exhibited increased accumulation in the tumor compared with their non-conjugated counterparts [147].

Wei et al. [156] reviewed the potential of aptamers as ligands for targeted drug delivery, mentioning another synergistic delivery system loading curcumin and gemcitabine functionalized with the AS1411 aptamer for pancreatic cancer therapy.

#### 5.4.2. Elemene

Elemene, a sesquiterpene compound derived from *Curcuma wenyujin* [157], is currently under investigation for its multifaceted pharmacological effects. Recent studies have unveiled its antioxidant, antiproliferative, and antitumor attributes [158]. β-elemene exerts its antitumor effects, encompassing apoptosis induction, cell cycle arrest, the inhibition of angiogenesis and cell migration, the augmentation of immunogenicity in tumor cells, and the suppression of CSC-like effects [158]. β-elemene not only directly combats tumors but also tackles multidrug resistance by diminishing mitochondrial membrane potential, activating the intracellular redox system, and prompting apoptosis in tumor cells. Moreover, β-elemene amplifies chemosensitivity by stimulating tumor cell apoptosis. These findings underscore the versatile potential of β-elemene in cancer treatment, positioning it as a promising therapeutic agent capable of exerting direct antitumor effects while overcoming challenges associated with treatment resistance [159]. It has been used as an adjuvant treatment for lung, gastric, and colorectal cancers [148].

In their previous work, Zhou and colleagues demonstrated a significant enhancement in the antiproliferative effect on colon cancer cells through the combined use of β-elemene and paclitaxel (PTX), surpassing their individual impacts [148]. The sequence of this aptamer consists of 48 oligonucleotides and adopts a hairpin secondary structure [160]. They developed a microemulsion functionalized with the SYL3C aptamer to deliver PTX and β-elemene to colorectal cancer cells. SYL3C is capable of targeting EpCAM, which is overexpressed in colorectal cancer cells [148]. To prepare the microemulsion, a one-step emulsion method was used. β-elemene and PTX were dissolved in a water-dispersible surfactant (1944 CS), and MAL-DSPE-PEG, HS15, DOPE, and PEG 400 were added to the mixture. Following this, the microemulsion was functionalized with the SYL3C aptamer, which is thiolated, through a thiol–maleimide reaction. The β-elemene and PTX microemulsion with SYL3C aptamer suppressed tumor growth, extended the survival of mice, and stimulated apoptosis in the cells within the tumor tissues. The enhanced anticancer efficacy is related to the downregulation of mutant p53, induction of M1 macrophage polarization, and reduction in bcl-2 expression and anti-apoptotic proteins [148].

#### 5.4.3. Luteolin

Luteolin, a flavone abundant in fruits, vegetables, and flowers, possesses anti-inflammatory, antihypertensive [161], and anticancer properties, which are related to its ability to induce apoptosis, inhibit proliferation, and prevent angiogenesis [162]. Notably, it modulates the JNK protein in cancer cells [163], suppresses anti-apoptotic proteins like Bcl-2 and Bcl-xL, activates caspases, and triggers DNA damage through ROS, thereby activating p53 [164]. Its broad-spectrum anticancer activity encompasses leukemia, where it exhibits antiproliferative effects on HL-60 cells [165].

To target acute myeloid leukemia cells, where the upregulation of both CD117 and transferrin receptors has been documented, Zhu et al. developed a binary drug delivery system comprising nanoparticles loaded with daunorubicin (DN) and adorned with the CD117 aptamer (Apt) and nanoparticles loaded with luteolin (LUT) and decorated with transferrin (Tf). To perform that, anionic nanoparticles decorated with transferrin and loaded with luteolin, along with cationic nanoparticles decorated with the aptamer and loaded with daunorubicin, were prepared separately. Subsequently, the Apt/Tf-Dn/LUT delivery system was obtained through self-assembly, facilitated by electronic interaction [149].

The modified amino group of CD 117 aptamer was covalently bound to the carboxylic group of PEG-COOH through the NHS protocol [149]. The sequence of this aptamer consists of 35 oligonucleotides and adopts a G-quadruplex structure [166].

The cytotoxicity assessment of the formulations was conducted using the HL-60 cell line, while the efficacy of tumor growth inhibition in vivo and the pharmacokinetic profile were evaluated by injecting cells from the same cell line into mice. Apt/Tf-Dn/LUT notably reduced cell viability. A similar trend was observed in terms of tumor volume reduction. These findings suggest a superior efficacy of Apt/Tf-Dn/LUT compared with the two free drugs, whether combined with the aptamer or Tf individually, underscoring the synergistic effect of the drugs and dual targeting. Furthermore, Apt/Tf-Dn/LUT exhibited enhanced tumor distribution compared with other formulations [149].

#### 5.4.4. Shikonin

Shikonin, extracted from *Lithospermum erythrorhizon*, shows promising therapeutic potential in cancer progression and development by influencing various cellular processes and is able to enhance the efficacy of some quimiotherapic agents [167]. However, despite its effectiveness, its hydrophobic nature and limited solubility, resulting in poor oral bioavailability, and its rapid clearance limits its use, which has led researchers to develop nanoformulations to overcome these aspects [168].

This compound induces apoptosis in glioma cancer cells through multiple mechanisms, including the generation of ROS, reduction of glutathione levels, disturbance of mitochondrial membrane potential, upregulation of p53, and cleavage of PARP. The PARP cleavage, an enzyme crucial for DNA repair and apoptotic regulation, implies the activation of apoptotic pathways [169]. In glioma, cellular markers like CD133 and CD44 play a crucial role in identifying and characterizing CSCs, a subset of tumor cells with self-renewal ability and resistance to standard treatments. Studies have shown that the serum levels of CD133 and CD44, reflecting CSC activity, are significantly associated with tumor metastasis, recurrence, and prognosis [170]. These markers not only help to assess tumor aggressiveness and predict disease progression but also serve as potential targets for targeted therapies. Understanding these cellular markers and their correlation with glioma progression is essential for developing more effective and personalized therapeutic strategies.

Wang et al. developed a hyaluronic acid (HA)-based microemulsion loaded with docetaxel (DTX) and shikonin (SKN) and functionalized with the AS1411 aptamer to target the U87 cell line, derived from a human glioblastoma tumor. HA-based drug delivery systems have shown promise in cancer treatment due to the high affinity between CD44-overexpressed receptors and HA. Initially, SKN and DTX were dissolved to form an SKN&DTX-co-loaded microemulsion. Subsequently, the microemulsion was functionalized with the AS1411 aptamer. The aptamer was covalently bound via a modified thiol group through a thiol–maleimide reaction to Mal-DSPEPEG. The cytotoxicity assays and apoptosis induction studies were conducted using U87 cells, while the biodistribution profile and in vivo efficacy were evaluated in mice. Additionally, human brain microvascular endothelial cells were used to assess the in vitro permeation of the blood–brain barrier. The results demonstrated increased internalization, apoptosis induction, and cytotoxicity against U87 cells, along with enhanced permeability into HBMEC cells based on an in vitro blood–brain barrier model. Furthermore, the formulation reduced the formation of U87/CSC spheroids and downregulated the expression levels of CD133 and CD44 [150].

The same author and other colleagues later developed another drug delivery system targeting glioma cancer cells (G422 cells) using different targeting ligands. They designed a microemulsion loaded with DTX and shikonin functionalized with the AS1411 aptamer and T7 peptide targeting nucleolin and transferrin receptors, respectively. Both brain tumor cells and blood–brain barrier (BBB) vascular endothelial cells express certain receptors, such as the transferrin receptor (TfR) and nucleolin. However, while exogenous Tf is commonly used as a targeting ligand, its efficacy is limited due to the competitive inhibition of specific binding to TfR and the potential loss of biofunctions. Therefore, Wang et al. utilized the T7 peptide, which specifically binds to TfR. This system comprised a microemulsion of DTX/SKN with Fe_3_O_4_ nanoparticles in the inner layer and decorated with AS1411 aptamer and T7 peptide. Fe_3_O_4_@T7/AS1411-SKN/DTX exhibited magnetism in the presence of an external magnetic field [151].

Regarding cellular studies, G422 cells were employed to assess cellular uptake, proliferation, and cytotoxicity in vitro. The functionalization of the delivery system significantly reduced the IC50 of DTX from 1.4 ug/mL to 0.8 ug/mL. Moreover, both T7-SKN/DTX and AS1411-SKN/DTX demonstrated substantially higher cellular uptake compared with SKN/DTX after 8 h of incubation. Notably, T7/AS1411-SKN/DTX induced significantly stronger apoptosis than T7-SKN/DTX-M, indicating that dual-ligand modifications enhance apoptotic effects. In terms of pharmacokinetics, tumor distribution, and efficacy in vivo, mice treated with Fe_3_O_4_@T7/AS1411/SKN&DTX-M exhibited the highest drug distribution and increased survival rates, with no notable toxicity observed (evaluated by weight changes). Additionally, changes in luminescence after the mice were treated with different formulations showed that treatment with Fe_3_O_4_@T7/AS1411-SKN/DTX significantly inhibited CD133+ and CD44+ cells within glioma segments, further underscoring the effectiveness of dual-ligand modification in glioma-specific inhibition. These results were attributed not only to the use of dual targeting but also to the magnetism generated by Fe_3_O_4_ particles, which, under an external magnetic field, could accumulate in the targeted organs [151].

#### 5.4.5. Silibinin

Shahidi and colleagues developed a synergistic approach by combining doxorubicin (DOX), a first-line chemotherapeutic agent, with silibinin, a natural compound, aiming to enhance the efficacy of HER2-positive breast cancer treatment while mitigating treatment-related toxicity [152].

Silibinin, a natural plant polyphenol renowned for its antioxidant and anticancer properties, has an impact on apoptosis, cell cycle progression, and autophagic pathways, showcasing the potential for designing more effective anticancer strategies [171].

In the mentioned study, carboxylated graphene oxide (cGO) served as a carrier, and the HB5 aptamer was the targeting agent since it has an affinity to HER2. This aptamer is composed of 86 nucleotides and is predicted to have a hairpin secondary structure [172]. The amino-modified HB5 was covalently bound to the carboxylic groups of cGO. Subsequently, Sili and DOX were simultaneously added to Apt-cGO, mixed, and allowed to rest for 48 h at room temperature. The SK-BR-3 cell line (HER2 positive) and MCF-7 (HER2 negative) were used. Apt-cGO-DOX-silibinin exhibited higher intracellular accumulation levels compared with free DOX and silibinin. Importantly, it demonstrated superior induction of apoptosis in the SK-BR-3 cell line, highlighting the selectivity of the aptamer formulation for this specific cancer cell type [152].

## 6. Aptamers and Semi-Synthetic Products Derived from Plants

As previously mentioned, several of the compounds used in clinical practice today are derived from natural sources, mainly from plants. Some of these compounds are well-known and widely used in clinical practice on a relatively large scale, requiring semi-synthetic processes for their production. The drug delivery systems discussed below are centered around these drugs, which, despite their widespread use in clinical practice, still face pharmacokinetic and toxicity challenges that need to be addressed. Consequently, some authors continue to work on new formulations and enhanced targeted delivery strategies.

### 6.1. Camptothecin—Aptamer Carriers

Camptothecin is a natural alkaloid isolated from the bark and stem of the *Camptotheca acuminata* tree, native to China and discovered in 1966 [173]. This compound was identified as an inhibitor of the enzyme topoisomerase I, a key therapeutic target in cancer treatment, and caught the attention of scientists due to its remarkable anticancer activity [174,175,176]. However, despite its effectiveness against various types of cancer, the clinical application of camptothecin was initially limited due to its low water solubility and the occurrence of side effects [177]. To overcome these challenges, researchers developed camptothecin analogs with improved pharmacological properties [178].

Three of these analogs—topotecan, irinotecan, and belotecan—have been approved by the United States Food and Drug Administration for use in chemotherapy [179]. Furthermore, camptothecin has been extensively explored as a parent molecule for the development of novel anticancer agents. Researchers have investigated different formulation strategies and structural modifications to enhance the efficacy and safety of camptothecin as a therapeutic agent.

Gao et al. conducted a study involving camptothecin derivatives and aptamers, focusing on two precursor compounds (P1 and P2) that exhibited excellent anticancer activity against HCT116 cells (human colon cancer cells). Three drug-aptamer conjugates (SSC-1, SSC-2, and SSC-3) were prepared from modules 8 and 10, and SSC-3 resulted from three units of CPT. Target cells (HCT116) and nonspecific cells (Ramos) were incubated with oligonucleotides to evaluate the in vitro cytotoxicity of the drug–aptamer conjugates (SSC-1, SSC-2, and SSC-3). Conjugation with aptamer provided improved water solubility to the camptothecin derivatives and reduced side effects through targeted delivery. Both SSC-1 and SSC-2 demonstrated excellent inhibitory activity against the target HCT116 cells, with IC50 values lower than those of the corresponding small molecule precursors P1 or P2. When Ramos cells were incubated with SSC-1 and P1 independently, SSC-1 showed less nonspecific toxicity than P1, and the cell viability rate of Ramos cells was higher than that of HCT116 cells incubated with the same drug at the same concentration. Similarly, when Ramos cells were incubated with SSC-2 and P2, the nonspecific toxicity of SSC-2 against Ramos cells was reduced, whereas P2 inhibited both Ramos and HCT116 cells at the same level without selectivity. SSC-3 did not show favorable IC50 results and, therefore, requires further modifications. The results indicated that the pharmaceutical moiety could be selectively delivered to HCT116 cells and released inside cells, inhibiting cell proliferation with selectivity [180].

### 6.2. Taxanes—Aptamer Carriers

Taxanes, diterpenoid chemotherapeutic agents, are widely used in treating various cancers [181,182,183,184]. The first taxane, paclitaxel, was isolated from the Pacific yew tree (*Taxus brevifolia*) in the 1960s in a sequence of a screening program conducted by the National Cancer Institute in the USA [185]. Paclitaxel induces apoptosis primarily through microtubule polymerization and stabilization, leading to cell cycle arrest and death [186]. Nowadays, it is obtained through semi-synthetic processes rather than being directly extracted from plants, allowing for a more sustainable production. Later, a French scientist obtained docetaxel through a semi-synthetic pathway starting from 10-deacetylbaccatin, and in 1996, it obtained FDA approval for advanced breast cancer and later for metastatic castration-resistant prostate cancer [187]. More recently, cabazitaxel has emerged as a second-generation semi-synthetic taxane following the preclinical screening of molecules derived from 10-deacetylbaccatin-III [188].

Despite their therapeutic efficacy, taxanes face challenges in clinical use due to their poor water solubility [189], significant toxicity [190], non-selective distribution, and limited ability to penetrate the blood–brain barrier efficiently [191], which restricts their effectiveness in treating brain tumors. To address these challenges, researchers have explored various formulation and delivery strategies to enhance bioavailability and tissue distribution and reduce systemic toxicity. Efforts to overcome these limitations involve the development of novel drug delivery systems aimed at enhancing solubility, improving targeted delivery to tumor sites, and minimizing off-target effects [192,193,194,195].

#### 6.2.1. Cabazitaxel

Concerning cabazitaxel, Cheng et al. developed nanoparticle-modified liposomes (LP) functionalized with the TLS1c aptamer, known for its high specificity for MEAR hepatoma cells. In vitro experiments focused on cellular uptake and cytotoxicity using three cell lines: Caco-2, HepG2, and MEAR. Apt-CBZ-LP exhibited higher cytotoxicity in MEAR cells compared with Caco-2 and HepG2 cells, and a significantly higher uptake of Apt-CBZ-LP was observed in MEAR cells compared with untargeted formulations. The results indicated that the viability of Caco-2 or HepG2 cells treated with Apt-CBZ-LP was significantly higher compared with CBZ-LP and the free drug. However, MEAR cells treated with Apt-CBZ-LP exhibited the highest levels of cytotoxicity compared with CBZ-LP, suggesting that the aptamer-modified formulation presented specificity for MEAR cells. In vivo toxicity was evaluated by monitoring changes in body weight in mice. Negative alterations were observed with the untargeted formulation, whereas no such effects were observed with Apt-CBZ-LP. Furthermore, Apt-CBZ-LP demonstrated significantly enhanced inhibitory effects on tumor growth compared with CBZ-LP or the free drug in the MEAR cell-induced tumor xenografts in mice. The biodistribution and antitumor efficacy analysis of Apt-CBZ-LP in vivo demonstrated the targeting of MEAR cells and specific localization within the tumor. These findings further support the initial hypothesis that the cytotoxicity of cabazitaxel decreases with aptamer-modified liposomes [196].

#### 6.2.2. Docetaxel

Fang et al. explored the potential of the Wy5a aptamer for targeting castration-resistant prostate cancer, focusing on PC-3 cells through the synthesis of DTX-loaded nanoparticles. Their objective encompassed the evaluation of internalization and cytotoxicity in vitro, along with the assessment of therapeutic efficacy and toxicity in vivo within xenograft models of prostate cancer. In vivo toxicity was evaluated by monitoring the white blood cell count. The results demonstrated that Apt-DTX-NP exhibited significantly higher cytotoxicity compared with their untargeted NPs and free DTX in vitro. Moreover, in vivo studies revealed that Apt-DTX-NP inhibited more effectively tumor growth compared with the untargeted nanoparticles and DTX alone. The white blood cell count remained within the normal range for mice treated with aptamer-modified and untargeted formulations, suggesting that the nanoparticles could mitigate the toxicity associated with DTX treatment [197]. Another interesting study was conducted by Yu et al. [198], who explored the use of the AS1411 aptamer for targeting nucleolin-expressing CT26 colon cancer cells using albumin nanoparticles loaded with DTX. Their objective was to evaluate cytotoxicity in vitro and tumor volume reduction in vivo by injecting a CT26 cell suspension into mice. The results showed that in vitro, Apt-NP-DTX exhibited stronger cytotoxicity compared with NP-DTX, specifically in CT26 cells. Conversely, both formulations demonstrated similar toxicity in CHO cells (cancer cell line from the ovary of the Chinese hamster), indicating that selectivity could be conferred by the aptamer. In vivo experiments demonstrated that the functionalized formulation resulted in the greatest reduction in tumor volume and extended survival rate in mice without causing changes in body weight, suggesting the absence of toxicity [198].

Wu et al. engineered A549-targeted lipid-polymer hybrid nanoparticles (NPs) encapsulating DTX and cisplatin (CIS). They evaluated in vitro cytotoxicity in A549 cells (NSCLC) and in vivo biodistribution and antitumor efficacy in xenografted mice. In vitro, aptamer-decorated APT-DTX/CIS-NP exhibited enhanced cell inhibition compared with other formulations. Notably, the dual drug-loaded NP showed superior cell inhibition compared with APT-DTX-NP and APT-CIS-NP, possibly attributed to the synergistic effect of both drugs co-encapsulated in the NP. Their findings revealed that in vivo, the uptake and accumulation of APT-DTX/CIS-NP were significantly higher than a non-modified NP, leading to superior tumor inhibition. NP distribution in the tumor was notably higher than with the free drug, which predominantly accumulated in the kidney and heart, revealing more toxicity. APT-DTX/CIS-NP also demonstrated enhanced tumor distribution compared with non-decorated DTX/CIS-NP, which was consistent with its superior antitumor efficacy [199]. By using aptamer-modified polymer nanoparticles (NPs) loaded with both drugs, Li et al. [200] investigated strategies to enhance the efficacy of DOX and DTX combination therapy. They specifically employed the Sgc8c aptamer to target the CCRF-CEM T lymphoblastoid cell line. Their study aimed to evaluate the uptake in tumor cells and the toxicity of the Apt-DOX/DTX-NP formulation. Their findings demonstrated that the combination of DOX and DTX exhibited a significantly lower IC50 compared with free DOX and free DTX, highlighting the synergistic effect of the drug combination. Various formulations were tested, including free DTX+DOX, DOX/DTX-NP, and Apt-DOX/DTX-NP. Notably, the Apt-DOX/DTX-NP formulation showed the most promising results in reducing the percentage of viable cells, indicating enhanced cytotoxicity. These results suggest that functionalized polymer nanoparticles loaded with DOX and DTX, guided by the Sgc8c aptamer, hold the potential to improve the efficacy of combination therapy in leukemia treatment [200].

Regarding possible treatments for melanomas, Zhang et al. [201] investigated the efficacy of targeted PLGA microcapsules with a core-shell structure incorporating DTX utilizing the PD-L1 aptamer to target B16 melanoma cells. Their study encompassed in vitro apoptotic studies on B16 cells and in vivo evaluations using a mouse melanoma model to assess the antitumor effect and toxicity in mice, measured by changes in body weight. In vitro experiments revealed higher apoptosis rates in the DTX-encapsulated microcapsule group (PLGA-DTX-PD-L1), which further increased over time. In vivo findings demonstrated that the combination of DTX and PD-L1 aptamer led to more pronounced antitumor effects. Specifically, the PLGA-DTX-PD-L1 microcapsules group exhibited the smallest tumor volume and slowest tumor growth rate. Moreover, the maintenance of body weight in mice indicated minimal toxicity associated with the treatment.

Concerning breast cancer, Ghassami et al. explored the efficacy of HER2-specific aptamer-guided nanoparticles in targeting SKOV-3 and MDA-MB-468 cell lines, representing HER2-positive and HER2-negative cell lines, respectively. Their study encompassed both in vitro and in vivo assessments, aiming to evaluate cytotoxicity, pharmacokinetics, and antitumor efficacy. In vitro cytotoxicity assays revealed that treatment with DTX-NP significantly decreased cell survival compared with the free drug. Moreover, Apt-DTX-NP demonstrated a significantly higher reduction in cell survival in the HER2-positive SKOV-3 cell line compared with untargeted DTX-NP. However, in the HER2-negative MDA-MB-468 cell line, no significant difference was observed between these two treatments. In vivo pharmacokinetic studies revealed that although the apparent volume of distribution did not significantly differ between Apt-DTX-NP and DTX-NP, Apt-DTX-NP prolonged the circulation of DTX in mice. Particularly in HER2-overexpressing tumor-bearing mice, treatment with Apt-DTX-NP resulted in a higher drug concentration at the tumor site compared with treatment with DTX-NP. Furthermore, in vivo antitumor efficacy assessments demonstrated the highest antitumor effect in the group treated with Apt-DTX-NP, indicating the superior therapeutic potential of HER2-specific aptamer-guided nanoparticles in targeting HER2-positive tumors [202].

Zolbanin et al. investigated the apoptotic effects of targeted co-delivery of DTX and c-Met siRNA (siMet) through MUC-1 aptamer-conjugated chitosan nanoparticles (NPs) on MUC-1-positive metastatic breast cancer cells (SKBR3) [203]. c-Met siRNA, a specific small interfering RNA (siRNA), was designed to silence the expression of the c-Met gene, a tyrosine kinase receptor crucial for cellular signaling pathways involved in cell proliferation, migration, invasion, and angiogenesis. The overexpression or hyperactivation of c-Met is common in many types of cancer, contributing to tumor progression and resistance to therapy [204]. Their experiments in vitro demonstrated that NPs containing siMet significantly increased apoptosis. In SKBR3 cells, the maximal effect was observed when cells were treated with targeted co-delivery NP + APT + siMet + DTX [203].

The same authors [205] later developed nanoparticles loaded with DTX and cMET siRNA to target MUC-1 overexpressing breast cancer cells, particularly SKBR3 cells. To evaluate the efficiency of mucin1 aptamer on nanoparticle uptake, Zolbanin et al. used SKBR3 cells (MUC-1 positive) and CHO cells (MUC-1 negative). Their findings demonstrated that the aptamer-modified formulation exhibited the highest uptake in SKBR3 cells and the least in CHO cells, confirming its specificity for MUC-1 positive. Additionally, the combination therapy system of DTX + siRNA loaded NPs showed better results in inhibiting cell viability compared with monotherapy with DTX-loaded NPs, indicating synergy between DTX and cMET siRNA. The aptamer-modified formulation also showed the highest reduction in the percentage of cell viability.

Additionally, for the treatment of breast cancer but with a focus on targeting MCF-7 cells, Kong et al. investigated the use of NPs loaded with DTX and functionalized with the AS1411 aptamer. In vitro experiments included studies on cellular uptake, drug release kinetics, cell viability, and antitumor efficacy in MCF-7 cells. In vivo assessments were conducted on antitumor efficacy in a xenograft breast tumor model. The results showed that modification and AS1411 functionalization did not alter the drug release properties of the prepared NP. However, the fluorescence intensity significantly increased in MCF-7 cells incubated with APT-DTX-NP, indicating high levels of in vitro cellular targeting efficacy due to the presence of AS1411 aptamers on the NP surface. Overall, the specific interactions between AS1411 aptamers and MCF-7 cells reinforced the endocytosis of APT-DTX-NP compared with untargeted NPs, resulting in better in vitro therapeutic efficacy for APT-DTX-NP compared with other formulations. In vivo, there were no differences observed in the body weight of mice across all NP treatment groups. However, the survival time of mice treated with APT-DTX-NP was higher compared with other groups. These results underscored the significant therapeutic efficacy of the target chemo-photothermal therapy strategy based on APT-DTX-NP [206]

#### 6.2.3. Paclitaxel

To target human MCF-7 (breast cancer cells) and Hela (cervical cancer cells), Guo et al. developed a spherical nucleic acid (SNA)-like micellar nanoparticle system loaded with PTX and functionalized with the AS1411 aptamer (AS1411/PTX-SNA). As previously mentioned, AS1411 targets nucleolin, which is overexpressed in the mentioned cell lines. The cell uptake efficiency of PTX-SNA, loaded with or without the aptamer, was assessed using MCF-7, HeLa, and normal L929 cells. Flow cytometry analysis revealed a higher fluorescence intensity in tumor cells treated with the AS1411/PTX-SNA, and there was no significant difference in cell uptake efficiency between AS1411/PTX-SNA and PTX-SNA in normal L929 cells, indicating enhanced targeting and cell entry facilitated by the aptamer. Apoptosis analysis conducted using MCF-7, HeLa, and L929 cells showed that AS1411/PTX-SNA exhibited the highest cytotoxicity against MCF-7 and HeLa cells compared with PTX-SNA and free PTX. Additionally, the viability of L929 cells remained unaffected by AS1411/PTX-SNA treatment, indicating the selectivity of aptamer-loaded PTX-SNAs for cancer cells. In vivo studies using MCF-7-cell-xenografted mice demonstrated that PTX-SNAs exhibited excellent tumor inhibition effects due to prolonged circulation time and enhancing tumor accumulation compared with free PTX. Remarkably, the targeting AS1411/PTX-SNA formulation showed superior performance compared with the nontargeting formulation. Importantly, no noticeable damage was observed in vital organs, such as the heart, liver, spleen, lung, and kidney, indicating low systemic toxicity of all PTX-SNA to mice [207]

Using the same breast cancer cell line (MCF-7) but with a different targeting approach, Mie et al. developed nanoparticles using elastin-like polypeptides (ELPs) fused with poly-aspartic acid chains (ELP-D) loaded with PTX. These nanoparticles were modified with the S2.2 aptamer to specifically deliver PTX to MCF-7 breast cancer cells, which overexpress the MUC1 protein on their surface. The formulations tested included PTX (carrier-free), PTX-ELP-D (carrier without aptamer), and PTX/ELP-D-Apt (carrier with aptamer). The results showed, through fluorescence microscopic images, greater cytotoxicity in the PTX/ELP-D-Apt formulation compared with the untargeted formulation. This suggests that the modification of the nanoparticles with the S2.2 aptamer, which has a affinity for MUC-1, increased the effectiveness of the PTX delivery system to MCF-7 breast cancer cells, resulting in increased MCF-7 cell death [208].

Also, for breast cancer therapeutics, more specifically TNBC, Duan et al. introduced a novel approach by developing PEGylated PLGA nanoparticles functionalized with the heparinase (HPA) aptamer (S1.5) (Apt-PTX-NP). The rationale behind targeting HPA lies in its overexpression in breast tumor tissues compared with normal breast tissue. By degrading heparan sulfate proteoglycans in the extracellular matrix, HPA facilitates the release of growth factors and cytokines that promote tumor progression. Therefore, Apt-PTX-NP was designed to specifically target HPA-expressing TNBC cells, aiming to inhibit tumor growth and metastasis. The objective of this study was to evaluate the in vitro cellular uptake and cytotoxicity of Apt-PTX-NP in MDA-MB-231 cells, a TNBC cell line. Additionally, the in vivo antitumor activity of the nanoparticles was assessed using mice bearing orthotopic MDA-MB-231 tumors. Toxicity was also evaluated based on changes in mice weight. Results showed that Apt-PTX-NP exhibited significantly higher cytotoxicity compared with PTX-NP and PTX alone in vitro. In vivo, Apt-PTX-NP demonstrated superior efficacy in reducing tumor size compared with PTX-NP and PTX. Importantly, the aptamer-modified nanoparticles exhibited minimal systemic toxicity, and mice tolerated all drug formulations extremely well, indicating their potential for clinical application with minimal side effects [209].

In turn, Wu et al. designed another drug delivery system loaded with paclitaxel to target MDA-MB-231 cells, employing a different targeting agent, the PD-L1 aptamer, due to the overexpression of PD-L1 protein in these cells. By expressing this protein, cancer cells can manipulate the immune system’s regulatory pathways to evade detection and destruction, enabling their survival and proliferation. This process is a significant focus of cancer immunotherapy research, with treatments aimed at blocking the PD-L1/PD-1 interaction to restore the immune system’s ability to recognize and eliminate cancer cells. The cytotoxicity was evaluated in vitro, and the results showed that the aptamer-PTX conjugate also exhibited enhanced cellular uptake and cytotoxicity in TNBC cells overexpressing PD-L1 [210].

Using the same cancer cell line, MDA-MB-231 and for the same purpose, Guo et al. developed a novel RNA four-way junction nanoparticle (4WJ) system designed for solubilizing and loading PTX for targeted cancer therapy. Functionalized with an EGFR aptamer, this nanoparticle system (PTX/4WJ-Apt) aimed to specifically target TNBC cells. The study focused on improving the solubility of PTX and reducing toxicity, which is another concern related to PTX. The results showed that the incorporation of PTX into RNA nanoparticles significantly enhanced its water solubility compared with free PTX. Animal trials conducted using an orthotopic TNBC xenograft tumors model in mice demonstrated that RNA nanoparticles incorporated with the anti-EGFR aptamer could specifically target tumors and effectively inhibit tumor growth. Moreover, these RNA-PTX nanoparticles exhibited minimal to undetectable toxicity and immune responses in mice. This study represents a significant advancement in addressing the insolubility of PTX, offering high-yield tumor-specific targeting and reduced adverse effects. Thus, it holds great potential for cancer therapy [211].

Kang et al. engineered lipid micellar nanoparticles containing quantum dots (QDs) and PTX (PTX-QDM) for imaging and treatment of colorectal cancer. They evaluated two targeting approaches to reach colorectal cancer cells: anti-EGFR antibodies (Ab) and anti-EGFR aptamers attached to the surface of PTX-QDM. The objective was to assess tumor distribution, tumor volume reduction, and toxicity evaluation by monitoring weight loss in mice bearing EGFR-positive LS174T tumor xenografts. The results indicated that both anti-EGFR antibodies and aptamers demonstrated similar targeting capacity, showing five times more targeting capacity than the untargeted carrier. Treatment with Ab-PTX-QDM or Apt-PTX-QDM effectively inhibited LS174T cell xenograft tumor growth at a relatively low dose of PTX. Mice treated with the Apt-PTX-QDM formulations did not exhibit significant weight loss, unlike those treated with free PTX, indicating the potential toxicity of free PTX. These results suggest that both Ab-QDM and Apt-QDM could serve as novel delivery vehicles for anticancer drugs, reducing side effects and enhancing therapeutic efficacy [212].

Also using the same target, EGFR, but to reach chordoma U-CH2 cells, that also overexpress this receptor, Xiao et al. developed a 3-way junction (3WJ) nanoparticle system functionalized with an EGFR aptamer and loaded with PTX. The efficacy of the drug once loaded was assessed. The Apt-PTX-3WJ nano system exhibited specificity for U-CH2 cells, unlike the non-functionalized 3WJ nanoparticles. Moreover, the aptamer-modified system demonstrated superior tumor cell inhibition compared with free PTX [213].

Shi et al. developed a novel Tetrahedral framework nucleic acid (tFNA) nanoparticle system for targeted drug delivery. Functionalized with two aptamers, GMT8, a short DNA sequence selected by SELEX, and Gint4.T, a 33 RNA oligonucleotide specific to PDGFRβ; this system aimed to target U87MG cells, a type of glioblastoma multiforme (GBM) cell line. PDGFRβ is frequently overexpressed in U87MG cells and plays a significant role in tumor progression, invasion, and treatment resistance in these tumors. Therefore, the high expression of platelet-derived growth factor receptor β (PDGFRβ) in U87MG cells makes them a relevant model for studying targeted therapies at this receptor in glioblastoma. The study focused on evaluating the accumulation of Apt-tFNA in cancer cells and its efficacy, as well as the effectiveness of the PTX/Apt-tFNA system. Results showed that Apt-tFNA accumulated substantially more effectively in U87MG and bEnd.3 cells compared with tFNA alone, likely due to the presence of the Gint4.T and GMT8 aptamers. Apt-tFNA remarkably suppressed U87MG cell proliferation, and when PTX was loaded into Apt-tFNA, cell viability was further reduced. Moreover, the system demonstrated significant inhibition of cell migration and invasion, two important characteristics of tumor cells. The study suggests that the aptamers alone have therapeutic potential, as Gint4.T notably inhibits the proliferation and migration of tumor cells by binding to PDGFRβ, inhibiting its activity and subsequent effects. However, when combined with PTX, the results of inhibiting cell migration and invasion were significantly superior compared with Apt-tFNA or PTX alone [214].

Engelberg et al. devised and investigated NPs composed of the biocompatible block-copolymer PEG-PCL entrapping the hydrophobic chemotherapeutic drug PTX. These micelles, modified with the aptamer s15, were designed to target human NSCLC cells. The selective cytotoxicity of APT-PTX-NP was evaluated in A549 target cells as well as in BEAS2B, HeLa, CaCo-2, FSE, and HEK-293 cells. The results demonstrated selective cytotoxicity of the APT-PTX-NP against target A549 cells, which further corroborated the confocal laser microscopy findings [215]. Chakraborty et al. investigated the efficacy of various malignant hepatocyte-targeting aptamers and other targeting agents, such as galactosamine and apotransferrin. Different formulations were tested, including free PTX, PTX-NP, and Apt-PTX-NP, alongside galactosamine and apotransferrin functionalized PTX-NP. Based on in vitro assessments like cytotoxicity, cell cycle analysis, and apoptotic potential in HCC cells (HepG2 and Huh-7), aptamer L5 was selected for further investigation. The ability of L5 to effectively interact with TAG-72 and HSP70, which are minimally expressed in normal hepatocytes but highly expressed in neoplastic hepatocytes, makes it a remarkably efficient ligand for targeting neoplastic hepatocytes. Results indicated that Apt-PTX-NP showed the most therapeutic potential among the tested formulations, with low toxicity. In vivo toxicological assessments were aligned with in vitro findings, as no significant changes in body weight were observed in mice treated with Apt-PTX-NP. The ability of aptamers L2 and L5 to interact effectively with TAG-72 and HSP70, highly expressed in neoplastic hepatocytes, makes them efficient ligands for preferentially targeting these cells. In vitro toxicity assessments in normal hepatocytes revealed that galactosamine and apotransferrin-modified formulations exhibited significantly higher toxicity compared with aptamer-functionalized nanoparticles [216].

## 7. Conclusions

In conclusion, the integration of aptamers into targeted drug delivery systems holds immense promise for overcoming the inherent challenges associated with natural compounds, namely plant-based compounds, in medical applications. The limitations of poor pharmacokinetics, low specificity, and potential toxicity often associated with natural products can be effectively addressed through the strategic use of aptamers as targeting agents. Aptamers, with their simplicity of synthesis and modification, high tissue permeability, stability, and a diverse range of available targets, emerge as powerful tools in enhancing the selectivity and effectiveness of natural compounds. By combining these aptamer-based delivery systems with natural products, a synergistic approach emerges, unlocking new possibilities and applications in the treatment of various conditions, especially in cancer treatment. As highlighted in this comprehensive review, numerous researchers have effectively employed this approach in their studies, yielding promising and improved outcomes compared with unconjugated systems. This review sheds light on the promising potential of aptamers, offering insights into their role as key players in advancing targeted drug delivery and opening avenues for continued exploration and innovation in the dynamic field of natural products.

## Figures and Tables

**Table 1:** Aptamer-based drug carriers.

Compound		Nanocarrier	Formulations	Aptamer	Oligonucleotide	Main structure	Target	Clinical Application	Main Results Using Aptamer-Based Drug Carriers	Ref.
Apigenin		PEGylated liposomes	Free apigenin Apigenin-LP Apigenin-PEG-LP Apt-Apigenin-PEG-LP	AS1411	5′-GGT GGT GGT GGT TGT GGT GGT GGT GG-3′	G-quadruplex	Nucleolin	Hepatocellular carcinoma	Higher cytotoxic potential Higher percentage of apoptosis Improved bioavailability Higher accumulation in the tumor site	[93]
Celastrol		PEGylated PAMAM dendrimers G5	Free Ce Ab-Ce-PAMAM Apt-Ce-PAMAM	SYL3C	5′-CAC TAC AGA GGT TGC GTC TGT CCC ACG TTG TCA TGG GGG GTT GGC CTG-3′	Hairpin	EpCAM	Colorectal Adenocarcinoma	Higher accumulation Enhanced intratumoral penetration Higher efficacy	[135]
Curcumin		PEGylated PAMAM G5 dendrimers loaded with AuNPs	Free CUR PEG-AuPAMAM-CUR Apt-PEG-AuPAMAM-CUR	MUC-1	5′-GCAGTTG ATCCTTTGGATACCCTGGTTTTTTTTTT-3′	Hairpin	MUC-1	Colorectal Adenocarcinoma	High reduction in tumor volume Improved survival rate of mice Higher cellular uptake and internalization Higher cytotoxicity	[106]
Curcumin		Human serum albumin nanoparticle	Free CUR HSANP-CUR Apt-HSANP-CUR	HB5	5′-AACCGCCCAAATCCCTAAGAGTCT GCACTTGTCATTTTGTATATGTATTTGGTTTTTGGCTCTCACAGACACACTA CACACGCACA-3′	Hairpin	HER2	Breast cancer	Increased curcumin cell uptake Higher cytotoxicity for cancer cells Less cytotoxicity for other cells	[109]
		Liposomes	Free CUR CUR-LP Apt-CUR-LP	A15	5ʹ-CCCUCCUACAUAGGG-3ʹ	Hairpin	CD133	Prostate cancer	Less hemolytic effect Higher cancer cell internalization Higher accumulation on cancer tissues Higher reduction in tumor size	[102]
Homoharringtonine		Polymeric nanoparticles	Free HHT Apt-HHT-POL	EGFR	5′-CGGCUUUGCCGCUAUAAUGCA CGGAUUUAAUCGCCGUAGAAAAGCAUGUCAAAGCCG-3′	Hairpin	EGFR	Lung cancer	Higher apoptotic levels Fewer toxic effects on liver function	[91]
α-Mangosteen		Lipid–polymer combinational nanoparticles	MG-POL Apt-MG-POL	CD44	5′-GAGATTCATCACGCGCATAGTCTTGGGACGGTGTTAAACGAAAGGGGACGACCGACTATGCGATGATGTCTTC-3′	Hairpin	CD44	Breast cancer	Higher reduction and disaggregation of tumor spheroids Higher global efficacy Reduced clearance	[129]
Morin		Liposomes	Free Morin Morin-LP Apt-AU@morin-LP	AS1411	5′-GGT GGT GGT GGT TGT GGT GGT GGT GG-3′	G-quadruplex	Nucleolin	Gastric cancer	Higher cytotoxicity Lower toxicity for non-cancerous cells Increased apoptotic potential Decrease in cancer cell density Reduction of tumor weight and size in mice Prolonged survival of mice	[134]
Thymoquinone		Nanodroplet	Free TQ TQ-ND Apt-TQ-ND	AS1411	5′-GGTGGTGGTGGTTGTGGTGGT GGTGG-3′	G-quadruplex	Nucleolin	Breast cancer	Cytotoxic potential was relatively the same for aptamer-modified and untargeted nanoemulsions	[142]
Triptolide		-	Free TP Apt-TP	AS1411	5′-GGTGGTGGTGGTTGTGGTGGT GGTGG-3′	G-quadruplex	Nucleolin	TNBC	Higher efficiency in inhibiting tumor growth and inducing apoptosis Less physical toxicity	[143]
Synergistic therapy	Curcumin and cabazitaxel	Lipid-polymer hybrid nanoparticles	Free CUR/CTX Apt-CTX-POL Apt-CUR/CTX-POL	A10-3.2	5′-GGGAGGACGA UGCGGAUCA GCCAUGUUUACG UCACUCCU-3′	Hairpin	PSMA	Prostate cancer	Superior cell inhibition (compared with individual cabazitaxel or curcumin) Increased accumulation in the tumor	[147]
	β-elemene and paclitaxel	Microemulsion	Free β-elemene/PTX Apt-ME-β-elemene/PTX	SYL3C	5′-CAC TAC AGA GGT TGC GTC TGT CCC ACG TTG TCA TGG GGG GTT GGC CTG-3′	Hairpin	EpCAM	Colorectal cancer	Superior tumor growth suppression Extended mice survival Higher apoptotic levels	[148]
	Luteolin and daunorubicin	Lipid-polymer hybrid nanoparticles *	Free Dn Free Lut Free Dn/Lut Apt-Dn Tf-Lut Apt-Dn/Lut Tf-Dn/Lut Apt/Tf-Dn/Lut	CD117	5′-GGGGCCGGGGC AAGGGGGGG GTACCGTGGTAGGAC-3′	G-quadruplex	CD117	Acute myeloid leukemia	Enhanced tumor distribution Higher cytotoxicity Superior tumor growth suppression with Apt/Tf-Dn/LUT NP	[149]
	Shikonin and docetaxel	Hyaluronic acid-based microemulsion	SKN/DTX Apt-SKN/DTX-ME	AS1411	5′-GGTGGTGGTGGT TGTGGTGGT GGTGG-3′	G-quadruplex	Nucleolin	Glioma	Increased cell uptake Higher apoptotic levels Increased cytotoxicity Enhanced permeability Enhanced brain-specific accumulation Superior tumor growth suppression Extended mice survival	[150]
		Microemulsion *	SKN/DTX T7-SKN/DTX AS1411-SKN/DTX T7/AS1411-SKN/DTX Fe_3_O_4_@T7/AS1411-SKN/DTX	AS1411	5′-GGTGGTGGTGGT TGTGGTGGT GGTGG-3′	G-quadruplex	Nucleolin	Glioma	Higher cellular uptake Stronger apoptosis Mice treated with Fe_3_O_4_@T7/AS1411/SKN&DTX-M exhibited the highest drug distribution and increased survival rates, with no notable toxicity observed Inhibition of CD133+ and CD44+ cells within glioma segments	[151]
	Silibinin and doxorubicin	Carboxylated graphene Oxide	Free FOX Free Sili cGO-DOX-Sili Apt-cGO-DOX/Sili	HB5	5′-(AACCGCCCAAATC (dNP)60CTACACACCCACA)-3′	Hairpin	HER2	Breast cancer	Higher cytotoxicity Higher internalization induced higher apoptotic levels	[152]

* In these studies, the drug delivery system was built with more than one targeting agent, resulting in improved outcomes. The main results reported pertain to the outcomes obtained when the system was functionalized with all ligands.

## Abbreviations

Ab—antibody; Apt—aptamer; Au—gold; Ce—celastrol; cGO—carboxylated graphene oxide; CTX—cabazitaxel; CUR—curcumin; Dn—daunorubicin; DOX—doxorubicin; EpCAM—Epithelial cell adhesion molecule; HHT—homoharringtonine; HSA—human serum albumin; LP-liposomes; Lut—lutein; MEM—microemulsion; MG—mangosteen; *MUC*-*1*—Mucin-1; ND—nanodroplet; PAMAM—Poly (amidoamine); PEG—polyethylene glycol; PM—polymeric micelle; POL—polymeric; PSMA—prostate-specific membrane antigen; PTX—paxlitaxel; Sili—silibilin; SKN—shikonin; Tf—transferrin; TNBC—triple negative breast cancer; TP—triptolide; TQ—thymoquinone.
